# A finite element computational framework coupling four-chamber heart mechanics with the systemic and pulmonary circulations

**DOI:** 10.21203/rs.3.rs-9204887/v1

**Published:** 2026-04-10

**Authors:** Vahid Ziaei-Rad, Sandra Hager, Kenneth S. Campbell, Jonathan F. Wenk, Lik-Chuan Lee

**Affiliations:** 1 Department of Mechanical Engineering, Michigan State University, East Lansing, MI, United States; 2 Division of Cardiovascular Medicine, Department of Physiology, University of Kentucky, Lexington, KY, United States; 3 Department of Mechanical and Aerospace Engineering, University of Kentucky, Lexington, KY, United States

**Keywords:** Four-chamber heart model, Patient-specific heart simulation, Whole-heart modeling, Digital twin cardiology, Cardiac mechanics

## Abstract

We present a computational four-chamber heart modeling framework that integrates a 3D finite element (FE) model of heart mechanics with a 0D model of the systemic and pulmonary circulations in a closed-loop system. The computational framework incorporates patient-specific geometry, rule-based myocardial fiber architecture, and nonlinear transversely isotropic tissue mechanics to simulate the full cardiac cycle. A bidirectional 3D–0D coupling strategy together with physiologic epicardial boundary conditions enables stable beat-to-beat simulations. Built on the open-source FEniCS platform with a residual-based stabilized mixed (P1–P1) FE formulation, the computational framework is able to produce pressure-volume loops of the four chambers and myocardial strain waveforms that are comparable to those measured in healthy humans. The framework is used to simulate inter-ventricular interactions arising from a reduction in contractility of the left ventricle (LV) and right ventricle (RV). A reduction in LV contractility produces a 4.9% decrease in RV peak pressure whereas a reduction in RV contractility produces a 20% decrease in LV peak pressure. The framework sets the foundation for patient-specific whole-heart simulations of cardiovascular diseases and treatments in future work.

## 1 Introduction

Computational modeling of cardiac mechanics is an important tool for understanding heart diseases and developing digital twins to personalize therapies. This tool has been applied to develop fundamental understanding of acute heart function [2, 5, 16, 24, 26, 30, 32], investigate the effects of heart diseases such as hypertrophic cardiomyopathy [18, 19] and myocardial infarction [10], as well as evaluating treatments such as left ventricular-assist devices [7] and cardiac resynchronization therapy [6, 25]. Most of these studies, however, are conducted using computational frameworks based on left-ventricular and/or biventricular heart geometry.

Although the omission of specific heart chambers in a computational model may be justified, it presents a limitation, as simplified models often neglect the significant influence that the deformation of one chamber has on the rest of the cardiac geometry. Currently, there are a limited number of anatomically and physiologically realistic four-chamber heart computational models [8, 12, 20, 34]. The scarcity of such models is attributed to difficulties in overcoming the geometric complexity of the whole-heart anatomy, the high computational cost of simulations, and various numerical challenges, particularly the instabilities arising at 3D–0D interfaces when coupled with models of the human circulation.

Existing four-chamber heart models, on the other hand, have several key limitations. *First*, while recent works have incorporated active atrial stress [12, 34], many whole-heart models still treat the atria as passive conduits [1, 24]. As such, these passive models are unable to consider the contribution to ventricular preload arising from atrial contraction. *Second*, existing whole-heart simulations typically employ only partial or one-way coupling with the systemic and pulmonary circulations—often through prescribed outlet pressures or other open-loop boundary conditions [12]. *Third*, existing models frequently use simplified boundary conditions—such as impedance-type or basic spring–dashpot formulations—that do not reproduce physiologic base–to–apex motion, with more realistic epicardial constraints emerging only recently [34]. *Last*, some four-chamber heart computational frameworks rely on monolithic solvers and extreme high-performance computational resources that limit their accessibility [12], though efficient segregated schemes have recently been proposed to address this issue [34].

Here, we seek to overcome these limitations by developing a finite element model of the four-chamber human heart integrated with the cardiopulmonary circulation in a comprehensive computational framework. This framework incorporates active atrial contraction and its interaction with the ventricles, enabling the reproduction of physiological measurements such as pressure–volume loops and myocardial strain waveforms. Furthermore, the model reproduces *in-vivo* physiologic heart motion by employing a tensorial Robin spring–damper boundary condition with distinct normal and tangential components. Built on an open and modular platform using the finite element library FEniCS, the framework is highly accessible and applicable to diverse patient-specific geometries and circulation models.

## 2 Methods

### 2.1 Geometry

**Fig. 1:**
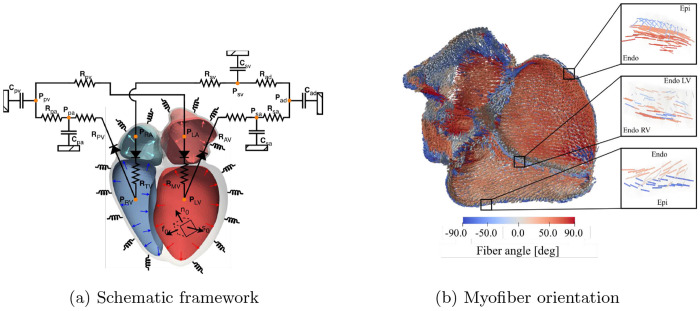
Overview of the modeling framework.

The geometry of the four-chamber heart model was derived from a dataset (case #1) in the publicly available virtual cohort of Niederer *et al* [29]. High-resolution whole-heart meshes from the dataset were post-processed. The post-processing steps include surface smoothing, mesh coarsening, and the creation of watertight chamber cavities. The resultant geometry preserves atrioventricular alignment, basal geometry, and valve plane fidelity. Ventricular fiber architecture was defined using a rule-based approach, with helix angles varying linearly across the wall from +60° at the endocardium to −60° at the epicardium [28]. Atrial fiber orientation was assigned using a mesh-independent, rule-based method from Wachter et al. [31], which is implemented in the open-source RESILIENT framework [4]. In that method, anatomical labels for atrial substructures, including the crista terminalis, Bachmann’s bundle, pectinate muscles, pulmonary vein sleeves, and appendages, were used to assign region-specific fiber directions from histological and diffusion tensor imaging data. Fiber fields were generated independent of the mesh by vector field interpolation and projected onto the FE mesh via barycentric coordinates and structural alignment. A schematic of the four-chamber heart model computational framework and a representative figure illustrating myofiber orientation and chamber boundaries, including regions where the boundary conditions were applied, are shown in Fig. 1.

### 2.2 Cardiac Mechanics Constitutive Model

Cardiac tissue mechanics is described by the first Piola–Kirchhoff stress tensor **P**, additively decomposed into passive and active parts:

$$P=P_{\text{passive}}+P_{\text{active}}.$$

The passive stress follows a transversely isotropic hyperelastic formulation adapted from a Fung-type model [11]. In that formulation, the strain energy density is

(1) $$W=\frac{1}{2}C\left(e^{Q}-1\right),$$

where *C* scales the passive myocardial stiffness. The variable *Q*, which captures the fiber–sheet–sheet-normal anisotropy, is defined by

(2) $$Q=b_{\mathrm{ff}}E_{\mathrm{ff}}^{2}+b_{\mathrm{xx}}(E_{\mathrm{ss}}^{2}+E_{\mathrm{nn}}^{2}+E_{\mathrm{sn}}^{2}+E_{\mathrm{ns}}^{2})\;+b_{\mathrm{fx}}(E_{\mathrm{fn}}^{2}+E_{\mathrm{nf}}^{2}+E_{\mathrm{fs}}^{2}+E_{\mathrm{sf}}^{2}),$$

with *E_ij_* the Green–Lagrange strain components in fiber (*f*), sheet (*s*), and sheet-normal (*n*) directions. Parameters *b_ff_*, *b_xx_*, and *b_fx_* control the anisotropic stiffness.

Active stress is modeled as time-dependent tension aligned with the myocardial fiber direction [7, 27]. The active component of **P**, which is oriented in the myofiber direction, is defined as

(3) $$P_{active}=T_{ref}\cdot \frac{Ca_{0}^{2}}{Ca_{0}^{2}+ECa_{50}^{2}}\cdot C_{t}(t)\cdot e_{f}⊗e_{f_{0}},$$

where *T*_ref_ is peak active tension, *Ca*_0_ the peak intracellular calcium concentration, and *ECa*_50_ is the length-dependent half-activation calcium threshold defined by

(4) $$ECa_{50}=\frac{{\left(Ca_{0}\right)}_{max}}{\sqrt{exp\left[B\left(l-l_{0}\right)\right]-1}}.$$

Here, *l* and *l*_0_ are the current and resting sarcomere lengths, respectively, and *B* governs length-dependent calcium sensitivity. Temporal activation is modeled by *C_t_*(*t*):

(5) $$C_{t}(t)=\{\begin{array}{ll} \frac{1}{2}\left[1-\text{cos}\left(\frac{\pi t}{t_{0}}\right)\right], & t<t_{t},\\ \frac{1}{2}\left[1-\text{cos}\left(\frac{\pi t_{t}}{t_{0}}\right)\right]\text{exp}\left(-\frac{t-t_{t}}{\tau }\right), & t\geq t_{t}, \end{array}$$

where *t*_0_ is time to peak active tension, *t_t_* the onset of relaxation, and *τ* the exponential decay time constant. The constitutive model parameters are given in Table 1.

### 2.3 Finite Element Formulation

The weak form of the momentum balance and material incompressibility constraint, incorporating passive and active myocardial mechanics, cavity pressure work, and epicardial spring–damper constraints is given by

(6) $$0=\int_{{\text{Ω}}_{0}}\left(P-pJF^{-T}\right):\nabla \delta udV-\int_{{\text{Ω}}_{0}}\delta p(J-1)dV\;+\int_{{\text{Γ}}_{\text{epi}}}\left(K_{\text{epi}}u+C_{\text{epi}}\dot{u}\right)\cdot \delta udS\;-\sum_{\text{ch}=\text{LA},\text{LV},\text{RA},\text{RV}}P_{\text{ch}}\int_{{\text{Γ}}_{\text{endo}}^{\text{ch}}}(\text{det}F)F^{-T}N\cdot \delta udS,$$

for all $\delta u\in H_{0}^{1}(\Omega_{0};\Gamma_{D})$ and *δ_p_* ∈ *L*^2^(Ω_0_). In Eq. (6), **P** = ∂*W*/∂**F** + **P**_active_ with **F** denoting the deformation gradient tensor, *p* is a Lagrange multiplier enforcing incompressibility, **u** is the displacement, **N** is the surface outward normal, and **K**_epi_, **C**_epi_ are symmetric positive-(semi) definite epicardial stiffness and damping tensors, respectively. Chamber pressures *P*_ch_ in Eq. (6) are supplied at each timestep by the solution of the closed-loop 0D circulatory model and applied as endocardial boundary conditions of the chamber in the FE formulation. The resulting cavity volumes from the FE solution are then returned (at each timestep) as input to the circulatory model, ensuring iterative pressure–volume consistency (see Section 2.4 for details).

#### 2.3.1 Stabilized Mixed Formulation

To mitigate volumetric locking and suppress spurious pressure oscillations inherent to an incompressible myocardium, we augment the weak form in Eq. (6) with a residual-based stabilization contribution acting on the pressure field. More precisely, the incompressibility part of Eq. (6),

$$-\int_{\Omega_{0}}\delta p(J-1)dV,$$

is replaced by

$$-\int_{\Omega_{0}}\delta p(J-1)dV+\sum_{e=1}^{n_{el}}\frac{\alpha \;h_{e}^{2}}{2\mu }\int_{\Omega_{e}}\nabla p\cdot \nabla \delta p\;dV,$$

where *h_e_* is the element size, *n*_el_ is the number of elements, *μ* is the shear modulus, and *α* = 𝒪(1). This stabilization remains a term within the weak formulation, rather than a separate governing equation, and enables stable equal-order interpolation and efficient large-scale simulations.

#### 2.3.2 Epicardial Boundary Condition

Pericardial restraint was modeled with a generalized Robin-type boundary condition applied to the heart epicardium, which resists radial displacement while allowing tangential sliding [23]. The boundary condition is given by

(7) $$PN+K_{epi}u+C_{epi}\frac{\partial u}{\partial t}=0,$$

where **P** is the first Piola–Kirchhoff stress tensor, **u** the displacement field, and **N** the outward surface normal of the epicardium. Anisotropic stiffness and damping tensors associated with the boundary condition were defined as:

(8a) $$K_{epi}=k_{n}\left(N⊗N\right)+k_{t}\left(I-N⊗N\right),$$

(8b) $$C_{epi}=c_{n}\left(N⊗N\right)+c_{t}\left(I-N⊗N\right),$$

with (*k_n_*, *k_t_*) and (*c_n_*, *c_t_*) denoting the (normal, tangential) stiffness and damping, respectively. The epicardial parameters were calibrated to match physiological chamber kinematics and pressure–volume loops with relatively small epicardial motion as observed *in vivo*. Additionally, a zero-displacement Dirichlet boundary condition was imposed on the truncated proximal aorta.

### 2.4 Modular Coupling of FE and Circulatory Models

The four-chamber 3D FE heart model was bidirectionally coupled to a closed-loop 0D circulatory model, with pressures and volumes exchanged at each timestep.

Specifically, volume–pressure residuals,

$$\vec{R}\left(P_{LV},P_{RV},P_{LA},P_{RA}\right)=\left[\begin{array}{c} V_{FE}^{LV}-V_{\text{circ}}^{LV}\\ V_{FE}^{RV}-V_{\text{circ}}^{RV}\\ V_{FE}^{LA}-V_{\text{circ}}^{LA}\\ V_{FE}^{RA}-V_{\text{circ}}^{RA} \end{array}\right],$$

were minimized ($\|\overset{\to }{R}\|\;<\;10^{-4}$) using a multivariate root-finding algorithm (fsolve in scipy) that iteratively adjusted chamber pressures until the difference between the FE-computed cavity volumes *V*_FE_^ch^ and the 0D-model cavity volumes *V*_circ_^ch^ for each chamber is below a prescribed tolerance. This modular 3D–0D scheme decouples cardiac mechanics and circulatory hemodynamic solvers, allowing substitution of alternative circulation models or numerical methods while preserving physiologic pressure–volume dynamics across cardiac cycles [13]. Details of the closed-loop four-chamber circulatory model, including vascular and valvular parameterization, are given in Appendix A.

The computational framework was implemented as an in-house FEM code built on the FEniCS platform (https://github.com/msucompbiolab/heArt/tree/heArt py3). The code supports modular boundary conditions, constitutive models, and circulatory coupling, and is maintained for reproducibility and scalability.

### 2.5 Simulation Cases

Using the coupled 3D-0D four-chamber heart computational framework, we first simulated a *baseline case* mimicking a healthy human heart. The heart wall was discretized with ~40K linear tetrahedral elements. Material and physiological parameters used in the model are given in Table 1. Multiple cardiac cycles were simulated until the coupled system reaches a time-periodic steady state. The model outputs included chamber pressure-volume (PV) loops, pressure waveforms, and regional strain distributions for analysis.

To probe ventricular–ventricular mechanical interdependence, an ischemic case was simulated by uniformly reducing active stress generation to 25% of its baseline value individually in the LV and RV while keeping all other parameters unchanged. This manipulation simulates cross-chamber effects through the shared interventricular septum and closed-loop circulation, enabling analysis of pressure attenuation and altered PV dynamics under contractile loss in individual chamber.

To characterize regional myocardial deformation in the simulation cases, longitudinal (*εu*) and circumferential (*ε*_*cc*_) strains were computed from the Green–Lagrange stretch tensor relative to the end-diastolic configuration. Strain was evaluated from the stretch ratios λ_*ij*_ along anatomically defined unit vectors **e**_*i*_ where *i* ∈ {*l, c*} (longitudinal, circumferential) [27]:

(9) $$\lambda_{ij}=\sqrt{e_{i}\cdot C\cdot e_{ij}},$$

where **C** = **F**^*T*^ **F** is the right Cauchy–Green tensor and **F** is the deformation gradient. Directional Euler–Almansi strain components were then defined as

(10) $$\varepsilon_{ij}=\frac{1}{2}\left(1-\frac{1}{\lambda_{ij}^{2}}\right)\times 100\%.$$

## 3 Results

### 3.1 Baseline case

**Fig. 2:**
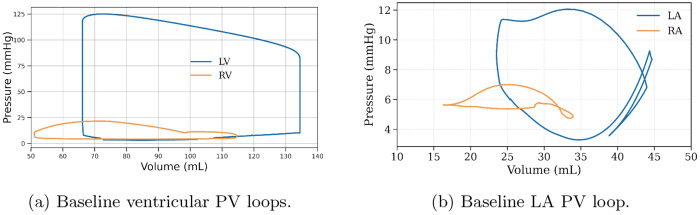
Baseline pressure–volume loops for ventricles (left) and left atrium (right).

Figure 2 shows the LV, RV, RA and LA PV loops over one cardiac cycle after reaching time-periodic steady state in the *baseline case* (See Table A1 for calibrated parameters). The LV PV loop shows an end-systolic volume of approximately 66 mL and an end-diastolic volume of approximately 135 mL, yielding a stroke volume of about 69 mL and an ejection fraction of about 51%. The LV peak pressure is approximately 125 mmHg. The RV PV loop shows an end-systolic volume of approximately 51 mL and an end-diastolic volume of approximately 115 mL, yielding a stroke volume of about 64 mL and an ejection fraction of about 56%. The RV peak pressure is approximately 22 mmHg. Unlike the LV and RV PV loops, the LA and RA PV loops exhibit a “figure of 8” pattern comprising of 2 loops, namely, the V-loop associated with passive atria and ventricular filling, and the A-loop associated with atria contraction.

**Fig. 3:**
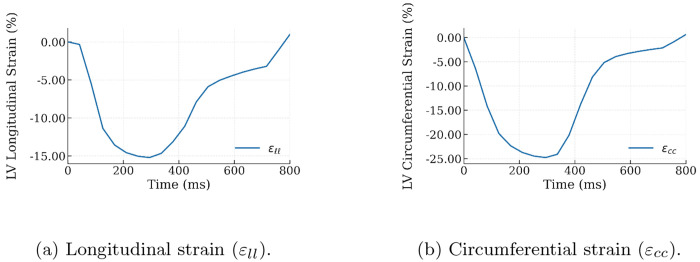
Global longitudinal and circumferential strain waveform of the LV in the baseline simulation.

Figure 3 shows the baseline global longitudinal (*ε_ll_*) and circumferential (*ε_cc_*) strain waveforms across the ventricles. Peak values of *ε_ll_* and *ε_cc_* are 15.2% and 24.8%, respectively. The time to peak value of *ε_ll_* and *ε_cc_* is 295 ms. The waveforms also show an increase in the strain rate at late diastole that is associated with atria contraction.

**Fig. 4:**
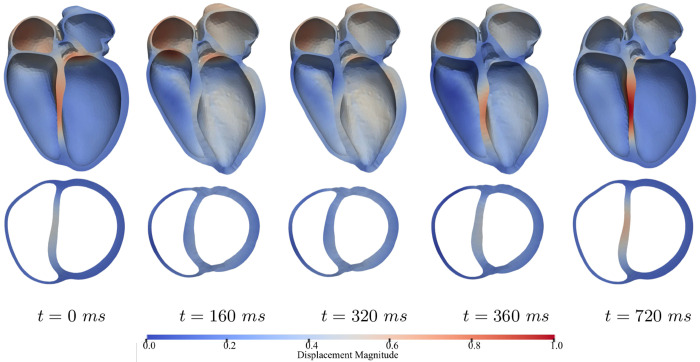
Spatiotemporal evolution of cardiac deformation over one cardiac cycle (T = 800 ms). Frames (left to right) correspond to key mechanical events: onset of LV contraction near mitral valve closure (t = 0 ms), peak LV pressure (t = 160 ms), RV filling onset near tricuspid valve opening (t = 320 ms), LV filling onset near mitral valve opening (t = 360 ms), and initiation of active atrial filling (t = 720 ms). The color bar denotes displacement magnitude.

Figure 4 shows the simulated deformation and motion of the heart over one full cardiac cycle at steady time-periodic state starting at end-diastole. The figure shows wall thickening and less motion in the apex compared to the base when the heart is contracting during systole.

### 3.2 Ischemia cases

**Fig. 5:**
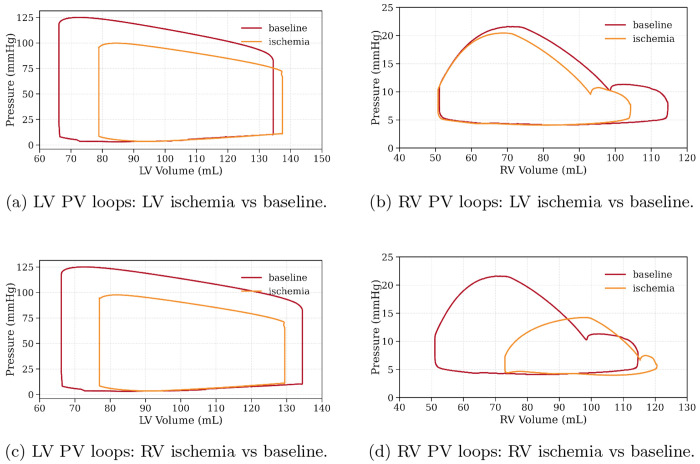
Baseline vs. ischemic comparisons for LV/RV PV loops.

Figure 5 shows the resulting LV and RV PV loops when either the LV or RV contractility was reduced to 0.25 of their values in the baseline case. When LV contractility was reduced, the LV peak pressure decreased from about 125 to 100 mmHg, while the RV peak pressure decreased from about 21.5 to 20.5 mmHg. In this case, the LV EF decreased from about 51% to 42%, and the RV EF decreased from about 56% to 51%. Conversely, when RV contractility was reduced, the LV peak pressure decreased from about 125 to 98 mmHg, while the RV peak pressure decreased from about 21.5 to 14.3 mmHg. For this case, the LV EF decreased from about 51% to 40%, and the RV EF decreased from about 56% to 40%.

### 3.3 Numerical Scalability

**Fig. 6:**
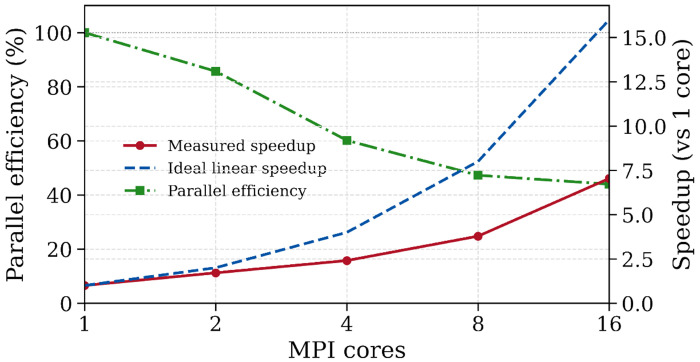
Scaling results with 40k tetrahedral elements, showing measured speedup and parallel efficiency.

Figure 6 shows the scaling results for the FEniCS implementation with MPI-based domain decomposition. Tests were performed on an agg-class AMD EPYC 9004 node (2×96 cores, ~750 GB RAM). A baseline run on 1 core required 37.5 s per timestep, whereas the 16-core configuration reduced this to 5.3 s, corresponding to an overall speedup of 7.0 and a parallel efficiency of 44.0% relative to 1 core. Parallel efficiency is near-ideal on 2 and 4 cores (85.7 and 60.2, respectively), but decreases to 47.3 and 44.0 on 8 and 16 cores as communication and linear-solver overheads become more pronounced. With 16 cores, the run time for a cardiac cycle is approximately 70 minutes.

## 4 Discussion

We have developed a computational modeling framework to simulate cardiac mechanics in a four-chamber heart that is coupled to 0D model of the systemic and pulmonary circulations in a closed-loop system. Using the computational framework, we were able to establish a baseline simulation case that can reproduce several key features found in the healthy human heart. Specifically, the simulated LV and RV PV loops have stroke volume (LV: 69 mL and RV: 64 mL), ejection fraction (LV: 51% and RV: 56%), peak pressure (LV: 125 mmHg; RV: 22 mmHg) that are comparable to those measured in the healthy human heart [15, 21]. The PV loops also show physiologic ventricular–arterial coupling where the steeper LV end-systolic limb relative to the RV indicated higher elastance, consistent with systemic versus pulmonary loading.

Besides the PV loop, the myocardial strains predicted by the computational framework are also comparable to 3D ECHO measurements of healthy human hearts. Specifically, the global LV longitudinal strain (*ε_ll_*) waveform showed absolute strain of 15%, which is comparable to the range (16 - 22%) measured in humans. Similarly, the global LV circumferential strain *ε_cc_* exhibits an absolute peak strain of approximately 25%, which is comparable to the range (21 - 28%) measured in humans [33].

Unlike four-chamber heart models that treat the atria as passive tissues [1, 24], we directly simulated this feature in the four-chamber computational framework. Atrial contraction plays an important role in reproducing physiological preload and ventricular filling. Its absence eliminates the A-loop from atrial pressure–volume curves and markedly reduces ventricular preload, producing nonphysiological behavior resembling atrial fibrillation [8]. By considering active atrial contraction, the framework produces a “figure of 8” pattern in the LA and RA PV loops and a steep increase in myocardial strains (i.e., “atrial kick) that resembles measurements found in the healthy human heart [14]. Similar to the LA PV loops measured by Khurram et al. [14], the area of the ‘V-loop’, which reflects the pulmonary venous inflow and mitral valve outflow in the passive atrium, is smaller than the ‘A-loop’ associated with atrial contraction.

The mechanics and motion of the whole-heart is critically affected by the boundary conditions imposed in the simulation. Detailed pericardial contact models—such as frictionless sliding or bidirectional penalty coupling to a pericardial reference body—can capture realistic epicardial motion [9]. These models, however, may require an auxiliary pericardial mesh, and often necessitate the fixation on vessel cutoffs when they are excluded. Many four-chamber heart models have instead constrained selected regions (e.g., base, apex, valve annuli) with spring–dashpot elements, or fix the great vessels leaving most of the epicardium traction-free, or applied omnidirectional springs that overly restrict tangential motion [3, 22]. Such simplifications, however, only partially reproduce physiologic pericardial–myocardial interaction. To overcome this issue, we prescribed an anisotropic Robin-type spring–dashpot formulation that acts primarily in the normal direction with minimal tangential resistance, similar to [8, 22]. This formulation is motivated by the fact that the pericardium’s mechanical influence arises from two components, namely, contact stress that limits radial expansion and augments ventricular interdependence, and lubrication associated with the pericardial fluid that permits low-friction tangential motion under normal attachment [22]. The implemented normal/tangential spring–dashpot boundary conditions preserves tangential slip, and enforces realistic pericardial restraint in a computationally scalable form [23]. The motion sequence in Fig. 4 is also consistent with this behavior, showing peak LV contraction near *t* = 160 ms, RV filling onset near *t* = 320 ms, LV filling onset near *t* = 360 ms, and active atrial filling near *t* = 720 ms. Modest damping imposed in the boundary conditions also helps stabilize beat-to-beat simulations by attenuating high-frequency oscillations [9].

### 4.1 Effects of changes in local myocardial contractility

Our simulations show that reducing RV contractility produces a larger percentage reduction in LV systolic pressure than the reciprocal manipulation. From Fig. 5, reducing LV contractility lowers RV peak pressure only from about 21.5 to 20.5 mmHg (about 4.7%) and changes RV EF from about 56% to 51%, whereas reducing RV contractility lowers LV peak pressure from about 125 to 98 mmHg (about 21.6%) and changes LV EF from about 51% to 40%. This directional asymmetry is consistent with the findings of Maughan et al. [17]. In that work, they measured systolic cross-talk gains to be approximately twofold larger from right-to-left than left-to-right using direct isovolumic perturbations (*G_R→L_* = 0.146 ± 0.030 vs. *G_L→R_* = 0.080 ± 0.013) and ESPVR-shift analysis (0.126 ± 0.016 vs. 0.065 ± 0.008). This ESPVR method showed leftward shifts without slope change in the output ventricle when the contralateral systolic pressure was increased, providing a mechanical basis for the larger LV pressure decrement when RV performance declines. It must be noted, however, that the measurements were performed in isolated heart preparation that considers ventricular-interdependence only through the septum while the simulation results also consider the interdependence occurring indirectly through the circulatory system.

### 4.2 Numerical challenges and comparison with other computational frameworks

Residual-based pressure stabilization in the incompressibility equation suppresses volumetric locking and pressure checkerboarding that arises when simulating the deformation of nearly and perfect incompressible material. Incorporating pressure stabilization enables equal-order (P1–P1) displacement–pressure interpolation, which is computationally less expensive, to be used. This approach permits discretizing the four-chamber heart with linear tetrahedra, which helps reduce the degrees of freedom and memory requirements as well as simplifies the thin-walled atrial geometry. The formulation remains robust when bidirectionally coupled to the closed-loop 0D circulatory model, with pressure–volume residuals iterated to preserve physiologic dynamics across beats. These properties make the stabilized P1–P1 method well suited for scalable, numerically stable whole-heart simulations.

Strong-scaling tests of the FEniCS-based solver show an order-of-magnitude reduction in wall time per timestep as the core count is increased, with parallel efficiency remaining high at low core counts and gradually decreasing as more cores are used. For this moderate P1–P1 mesh with roughly 40k elements, the onset of diminishing returns beyond about 4–8 cores is consistent with the expected strong-scaling limit, once only a few thousand elements remain per core and communication and global linear-solver operations start to dominate. This behavior also motivates restricting the present benchmarks to 16 cores, which already probe the regime where overheads become comparable to local computation. We expect the same implementation to maintain good parallel efficiency at substantially higher core counts for problems with finer meshes and more degrees of freedom per rank.

## 5 Limitations

This study has several limitations. *First*, the geometry was post-processed to create water-tight chambers and a mesh with reduced complexities. These modifications suppress small anatomical features and trabeculations that can bias the cavity volume, local curvature, and wall stress. They may also constrain annular motion to the discretized surface. Results should therefore be interpreted at chamber and regional scales rather than at the level of substructures such as papillary muscles or valve apparatus. *Second*, there are some simplifications made in terms of physiology and description of hemodynamics. Particularly, ventricular and atrial fibers were prescribed using a rule-based approach, not based on sub ject-specific data. *Third*, the active contraction behavior was described based on a phenomenological tension model, limiting fidelity for load-, rate-, and history-dependent behaviors. Heart valves leaflet dynamics and blood flow are also not mechanically modeled.

## 6 Conclusion

We developed an integrated four-chamber finite-element electromechanics model that couples patient-specific anatomy and rule-based fiber architecture to a closed-loop systemic–pulmonary circulation through a pressure–volume residual minimization scheme. A stabilized mixed P1–P1 formulation and an anisotropic Robin epicardial spring–damper boundary condition enabled numerically stable beat-to-beat simulations, preserving physiologic chamber kinematics and PV dynamics. The framework reproduced physiological PV loops in the 4 chambers, myocardial strain waveforms as well as ventricular interdependence measured in experiments. The implementation also showed good parallel efficiency up to moderate core counts, supporting scalable whole-heart simulation on practical meshes. These results, together with the modular 3D–0D architecture, position the computational framework for hypothesis testing and patient-specific simulations in future studies.

## Figures and Tables

**Table 1:** Material and physiological parameters used in the constitutive model [27].

Symbol	Description	Value
*C*	Passive stiffness scaling coefficient [kPa]	130.0
*b_ff_*	Fiber-direction stiffness coefficient [–]	29.0
*b_xx_*	Cross-fiber/sheet stiffness coefficient [–]	26.6
*b_fx_*	Fiber–sheet shear stiffness coefficient [–]	13.3
*T*_ref_	Peak active fiber tension [kPa]	700.0
*Ca*_0_	Peak intracellular calcium concentration [μM]	4.35
(*Ca*_0_)_max_	Calcium concentration at half-activation [μM]	4.35
*B*	Sarcomere length–tension sensitivity [μm^−1^]	4.75
*l*_0_	Resting sarcomere length [μm]	1.58
*t*_0_	Time to peak active tension [ms]	275
*t_t_*	Onset time of relaxation [ms]	300
*τ*	Tension decay time constant [ms]	25

## Data Availability

The simulation data supporting the findings of this study are available from the corresponding author upon reasonable request.

## Article Highlights.

- Patient-specific four-chamber heart mechanics is coupled to closed-loop systemic and pulmonary circulation.
- Stabilized P1–P1 finite elements and epicardial spring–damper conditions enable robust whole-heart simulation.
- Simulations reproduce physiologic chamber dynamics and reveal stronger RV-to-LV pressure coupling than the reverse.

## Appendix A Closed-Loop Four-Chamber Circulatory Model

The 0D model comprises resistive–compliant vascular compartments and pressure-driven unidirectional valves, with parameter values listed in Table A1. The systemic loop includes proximal/distal aorta (*V_sa_, V_ad_*) and systemic veins (*V_sv_*); the pulmonary loop includes pulmonary arteries (*V_pa_*) and veins (*V_pv_*). Volume conservation is enforced, with FE chamber volumes exchanged at each timestep.

Mitral, aortic, tricuspid, and pulmonary valves are ideal one-way elements, open when upstream pressure exceeds downstream:

(A1a)
$$q_{mv}=max\left(0,\frac{P_{LA}-P_{LV}}{R_{mv}}\right),\;q_{av}=max\left(0,\frac{P_{LV}-P_{sa}}{R_{av}}\right),$$

(A1b)
$$q_{tv}=max\left(0,\frac{P_{RA}-P_{RV}}{R_{tv}}\right),\;q_{pvv}=max\left(0,\frac{P_{RV}-P_{pa}}{R_{pvv}}\right).$$

**Table A1:** Lumped-parameter values in the closed-loop model. Parameters were automatically optimized to match healthy adult resting pressure–volume loops, peak pressures, and cardiac output.

Parameter	Description	Unit	Value
**Systemic Circulation**			
*R*_mv_	Mitral valve resistance	dyn·s/cm^5^	200.0
*R*_av_	Aortic valve resistance	dyn·s/cm^5^	500.0
*R*_sa_	Proximal aorta resistance	dyn·s/cm^5^	18000
*R*_ad_	Distal aorta resistance	dyn·s/cm^5^	31800
*R*_sv_	Systemic venous resistance	dyn·s/cm^5^	100.0
*C*_sa_	Proximal aorta compliance	mL/mmHg	0.0055
*C*_ad_	Distal aorta compliance	mL/mmHg	0.033
*C*_sv_	Venous compliance	mL/mmHg	0.28
*V*_sa_,0	Unstressed proximal aorta volume	mL	550
*V*_ad_,0	Unstressed distal aorta volume	mL	40
*V*_sv_,0	Unstressed venous volume	mL	2550.0

**Pulmonary Circulation**			
*R*_tv_	Tricuspid valve resistance	dyn·s/cm^5^	800.0
*R*_pvv_	Pulmonary valve resistance	dyn·s/cm^5^	400.0
*R*_pa_	Pulmonary artery resistance	dyn·s/cm^5^	10000.0
*R*_pv_	Pulmonary vein resistance	dyn·s/cm^5^	500.0
*C*_pa_	Pulmonary artery compliance	mL/mmHg	0.01
*C*_pv_	Pulmonary vein compliance	mL/mmHg	0.9
*V*_pa_, 0	Unstressed pulmonary artery volume	mL	320
*V*_pv_,0	Unstressed pulmonary vein volume	mL	2000

**Atrial Activation**			
*T*_max,la/ra_	τ Peak contraction time	ms	120 / 120
*τ*_la/ra_	Relaxation time constant	ms	25 / 25
tdelay_la/ra_	Activation delay	ms	220 / 160

**Initial Compartment Volumes**			
*V*_sv_	Systemic veins	mL	2600.0
*V*_sa_	Proximal aorta	mL	739.0
*V*_ad_	Distal aorta	mL	283.0
*V*_pv_	Pulmonary veins	mL	3829.0
*V*_pa_	Pulmonary arteries	mL	402.0

Systemic and pulmonary vessels follow linear resistance laws:

(A2a)
$$q_{sa}=\frac{P_{sa}-P_{ad}}{R_{sa}},\;q_{ad}=\frac{P_{ad}-P_{sv}}{R_{ad}},\;q_{sv}=\frac{P_{sv}-P_{RA}}{R_{sv}},$$

(A2b)
$$q_{pa}=\frac{P_{pa}-P_{pv}}{R_{pa}},\;q_{pv}=\frac{P_{pv}-P_{LA}}{R_{pv}}.$$

Pressures are computed from compartment compliance:

(A3)
$$P_{i}=\frac{V_{i}-V_{i,0}}{C_{i}},\;i\in \{sa,ad,sv,pa,pv\}.$$

Volumes are updated by explicit Euler integration:

(A4a)
$$V_{LV}^{t+\Delta t}=V_{LV}^{t}+\Delta t\left(q_{mv}-q_{av}\right),\;V_{sa}^{t+\Delta t}=V_{sa}^{t}+\Delta t\left(q_{av}-q_{sa}\right),$$

(A4b)
$$V_{ad}^{t+\Delta t}=V_{ad}^{t}+\Delta t\left(q_{sa}-q_{ad}\right),\;\;V_{sv}^{t+\Delta t}=V_{sv}^{t}+\Delta t\left(q_{ad}-q_{sv}\right),$$

(A4c)
$$V_{RA}^{t+\Delta t}=V_{RA}^{t}+\Delta t\left(q_{sv}-q_{tv}\right),\;\;V_{RV}^{t+\Delta t}=V_{RV}^{t}+\Delta t\left(q_{tv}-q_{pvv}\right),$$

(A4d)
$$V_{pa}^{t+\Delta t}=V_{pa}^{t}+\Delta t\left(q_{pvv}-q_{pa}\right),\;\;V_{pv}^{t+\Delta t}=V_{pv}^{t}+\Delta t\left(q_{pa}-q_{pv}\right),$$

(A4e)
$$V_{LA}^{t+\Delta t}=V_{LA}^{t}+\Delta t\left(q_{pv}-q_{mv}\right).$$

## Code availability:

The computational framework used in this study is available at https://github.com/msucompbiolab/heArt/tree/heArt_py3.
